# Quantification of Sesquiterpene Lactones in Asteraceae Plant Extracts: Evaluation of their Allergenic Potential

**DOI:** 10.3797/scipharm.1306-17

**Published:** 2013-08-15

**Authors:** Helena Salapovic, Johannes Geier, Gottfried Reznicek

**Affiliations:** 1 Department of Pharmacognosy, University of Vienna, Austria.; 2 Information Network of Departments of Dermatology, University of Göttingen, Germany.

**Keywords:** Quantitative determination of sesquiterpene lactones, Allergenic potential of Asteraceae plants, Compositae mix, Allergic contact dermatitis, Alpha-methylene-gamma-lactone

## Abstract

Sesquiterpene lactones (SLs), mainly those with an activated exocyclic methylene group, are important allergens in Asteraceae (Compositae) plants. As a screening tool, the Compositae mix, consisting of five Asteraceae plant extracts with allergenic potential (feverfew, tansy, arnica, yarrow, and German chamomile) is part of several national patch test baseline series. However, the SL content of the Compositae mix may vary due to the source material. Therefore, a simple spectrophotometric method for the quantitative measurement of SLs with the α-methylene-γ-butyrolactone moiety was developed, giving the percentage of allergenic compounds in plant extracts. The method has been validated and five Asteraceae extracts, namely feverfew (*Tanacetum parthenium* L.), tansy (*Tanacetum vulgare* L.), arnica (*Arnica montana* L.), yarrow (*Achillea millefolium* L.), and German chamomile (*Chamomilla recutita* L. Rauschert) that have been used in routine patch test screening were evaluated. A good correlation could be found between the results obtained using the proposed spectrophotometric method and the corresponding clinical results. Thus, the introduced method is a valuable tool for evaluating the allergenic potential and for the simple and efficient quality control of plant extracts with allergenic potential.

## Introduction

**Fig. 1:**
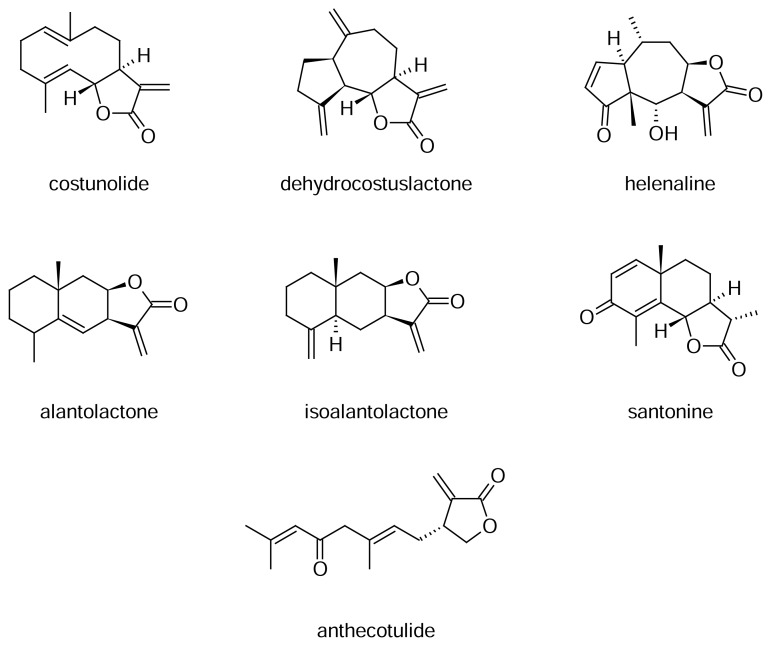
Chemical structures of selected sesquiterpene lactones

Sesquiterpene lactones (SLs) with an α-methylene group on the γ-lactone ring (Fig. 1) were identified as the main contact allergens in the Asteraceae (Compositae) family, one of the largest flowering plant families in the world [1]. Skin exposure to SLs through contact with plants or topical application of products like herbal medicines or cosmetic/skin care products containing Compositae extracts may cause sensitization to SLs resulting in allergic contact dermatitis [2, 3]. The SL mix and the Compositae mix have been used as screening tools for SL allergies in clinically conducted patch tests [4]. The SL mix 0.1% petrolatum (pet.) contains clearly defined equal parts of alantolactone, costunolide, and dehydrocostuslactone (Fig. 1). In contrast, the SL content of the Compositae mix has not yet been standardized nor sufficiently controlled. Varying SL contents of the plant extracts applied for the production of the Compositae mix can be expected. This may contribute to observed differences in the patch test results from applying the Compositae mix, apart from other sensitizers present in the Compositae mix (e.g. polyacetylenes).

A general qualitative allergen-proving analytical method using thin-layer chromatography has been shown to be insufficient for the quality control of the extracts. Thus, a reliable method for the quantification of SLs in Compositae mix is required [1, 5].

Some attempts have been made to determine allergens by chemical means. It could be shown that various pure, potentially allergenic compounds react selectively with nucleophilic sulfhydryl (-SH) groups [1]. Amino acid derivatives, peptides, and proteins were used for the reaction with allergens [6], and their adducts were determined by high-performance liquid chromatography (HPLC) [7] or matrix-assisted laser desorption mass spectrometry (MALDI-MS) [8]. Also, non-peptide/protein models were applied for the selective reaction with allergens [9], however, all these experiments were carried out with pure compounds. For the investigation of plant extracts, gas chromatography-mass spectrometry (GC-MS) as well as HPLC methods were proposed [10]. A disadvantage of these methods is that they are expensive and the allergenic compounds have to be known, otherwise no quantitative determination is possible.

Therefore, the aim of this work was to develop a suitable and simple method for the quantitative determination of SLs with an α-methylene-γ-butyrolactone moiety, representing the strongest allergens in Compositae. This tool should allow for the evaluation of the allergenic potential of plant extracts used for patch testing of SL-hypersensitivity.

## Results

### Principle of the Spectrophotometric Method

**Fig. 2:**
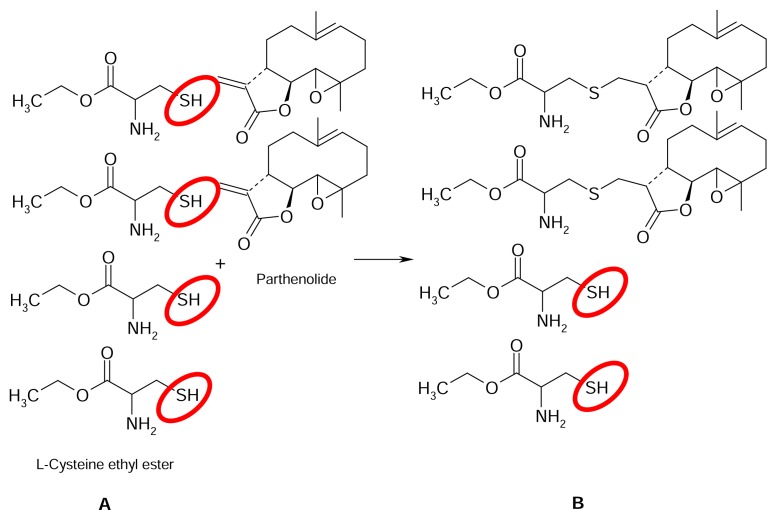
Scheme of allergen quantification by UV/VIS-spectrophotometry using the example of parthenolide (A: quantification of free SH-groups using Ellman’s reagent before reaction with allergen. B: quantification of free SH-groups after reaction with allergen).

SLs with an α-methylene-γ-butyrolactone-containing moiety react through a Michael-addition with free sulfhydryl- (-SH) or amino- (-NH_2_) groups in proteins after penetration into the skin. This reaction is probably the very first step in contact sensitization to SLs. This principle of allergen-binding is counterfeited in the present work by carrying out the reaction under alkaline conditions. The SL parthenolide with the typical α-methylene-γ-butyrolactone moiety and L-Cysteine ethylester hydrochloride (CE) with a free sulfhydryl group (Fig. 2, A) were chosen as model substances for the method development to react and give the Michael-addition product (Fig. 2, B). The remaining free SH-groups of CE after the reaction (Fig. 2, B) could easily be quantified by UV/VIS-spectrophotometry using Ellman’s reagent [11]. Thus, the principle of the method is an indirect determination of the remaining free SH-groups with Ellman’s reagent after addition of a reactive SL with an excess of L-Cysteine ethylester hydrochloride (CE). The formation of an adduct between CE and parthenolide, as well as other allergenic SLs (e.g. alantolactone, anthecotulide, costunolide, dehydrocostuslactone, helenaline, and isoalantolactone), was confirmed by mass spectrometry. The respective molecular ion of the CE-SL-adduct could be detected for each tested SL. On the contrary, no CE-SL-adduct was observed when applying SLs without the reactive α-methylene group (e.g. santonine, Fig. 1).

### Linearity of Parthenolide

Five different concentrations (increasing aliquots 25–180 μl) of a stock solution of parthenolide were prepared and measured (n = 5) as described in “Experimental”. The obtained absorption values were plotted against the concentration of parthenolide and the linearity was determined using linear regression analysis. The linear range of parthenolide was found to be 11–82 μmol/l and the calibration curve y = −0.009x + 0.9981 gave a good fit with a correlation coefficient (R^2^) of 0.9994.

### Precision

The intra- and interday precisions (expressed as the percentage of the relative standard deviation, % RSD) of the allergen concentrations were evaluated using three concentration levels of parthenolide within the linear calibration range. Three replicates each of the freshly prepared parthenolide samples within one day and over five successive days were determined for the calculation of the precision of the method (Tab. 1).

### Measurement of Pure SLs

The reliability of the proposed spectrophotometric method was proven by measuring six other allergenic SLs containing an α-methylene-γ-butyrolactone moiety (Fig. 1). Freshly prepared solutions of each SL (five concentration levels each) were analyzed in triplicate continuously during the next six days. The linear concentration ranges of alantolactone, anthecotulide, costunolide, dehydrocostuslactone, and isoalantolactone were similar to that of parthenolide and the precisions varied from 1.00 to 2.96% RSD. The linear concentration range of helenaline was found to be 14–35 μmol/l and the precision varied between 1.42 and 2.31% RSD.

### Selectivity

In order to verify the selectivity of the method, the SL santonine was spectro-photometrically analyzed as well. An important structural characteristic of this compound is the absence of the α-methylene group on the γ-lactone ring (Fig. 1). After the reaction of santonine with CE, no adduct could be observed, neither with UV/VIS-spectrophotometry nor with mass spectrometry.

### Accuracy

To evaluate the accuracy of the method, extracts of yarrow (low allergen content) and tansy (high allergen content) were selected to investigate the possible matrix effects on the proposed analytical method.

Equal volumes (100 μL) of a freshly-prepared stock solution of yarrow extract were spiked with five different concentrations of parthenolide (11–82 μmol/l) and left at room temperature for 15 min. All spiked samples were measured against the original yarrow extract in triplicate to calculate the precision and the recovery rate. The precision (% RSD) and recovery (% R) of parthenolide were determined to be 1.27–2.98% RSD and 96.97–98.59% R.

The same procedure was chosen for spiking the tansy extract, where five different concentrations of parthenolide (14–64 μmol/l) were added to equal volumes (20 μl) of the extract stock solution and analyzed in triplicate in comparison to the original extract. The calculated precision (% RSD) and recovery (% R) of parthenolide were 1.18–2.88% RSD and 96.22–103.62% R.

### Determination of Plant Extracts

**Fig. 3:**
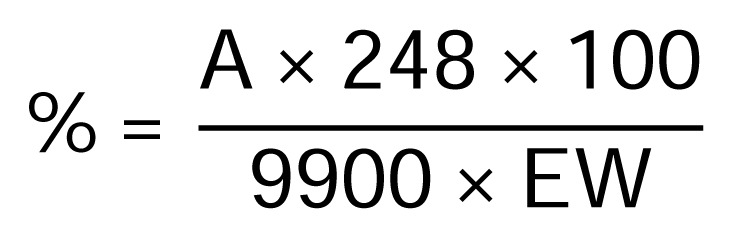
Formula for the calculation of % of allergenic sesquiterpene lactones. A is the absorbance (A_2_–A_1_, see Experimental) of the test solutions at 412 nm; EW is the weight of extract (g) regarding to 1000 ml. The molar absorption coefficient of L-cysteine ethyl ester hydrochloride after reaction with Ellman’s reagent was determined to be 9900.

**Fig. 4:**
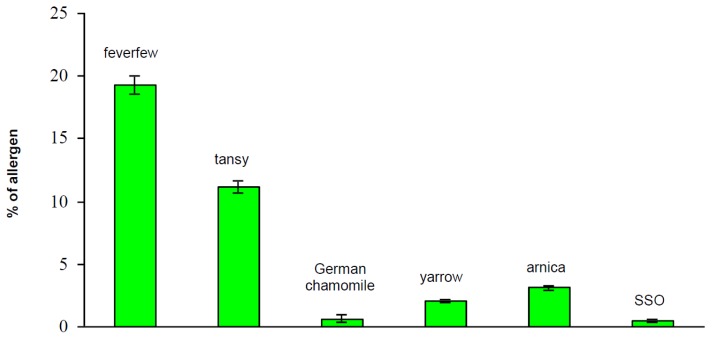
Percentage of allergens (calculated as parthenolide) in the individual ingredients of the Compositae mix as well as sorbitane sesquioleate by UV/VIS-spectrophotometry

In order to determine the amount of allergens in the plant extracts, the spectrophotometric method was applied to test individual ingredients of the Compositae mix. The five Asteraceae extracts were used for diagnostic patch testing. The spectrophotometric measurements (n = 6, except German chamomile n = 9) of each extract solution were carried out in triplicate. The percentage (%) of allergens were calculated using the formula (Fig. 3) and the obtained data, expressed as the mean value of the single measurements, the standard deviations (s), and the relative standard deviations (% RSD) for each sample extract are given in Fig. 4. A solution of 20% sorbitane sesquioleate (SSO, this agent was used as an emulsifier to prepare the extract solutions) was also tested (n = 8) with the proposed spectrophotometric method.

Reanalyzing other extract samples from the same batch provided by Hermal one year later confirmed the excellent reproducibility of the given method, as no significant differences in the results of these two series could be detected. Moreover, a mixture of the five crude extracts (each 10.0 ± 0.1 mg) was prepared, dissolved in 1 ml methanol/dichloromethane (1/1, v/v) and analyzed spectrophotometrically. The results obtained for the three independently prepared mixtures were 6.51%, 6.23%, and 6.33%, respectively, pointing out that the data are close to the mean value calculated from the analyses of the single extracts (see Fig. 4, mean value = 7.33%).

### Patch Test Results of Individual Plant Extracts and their Mixes

**Fig. 5:**
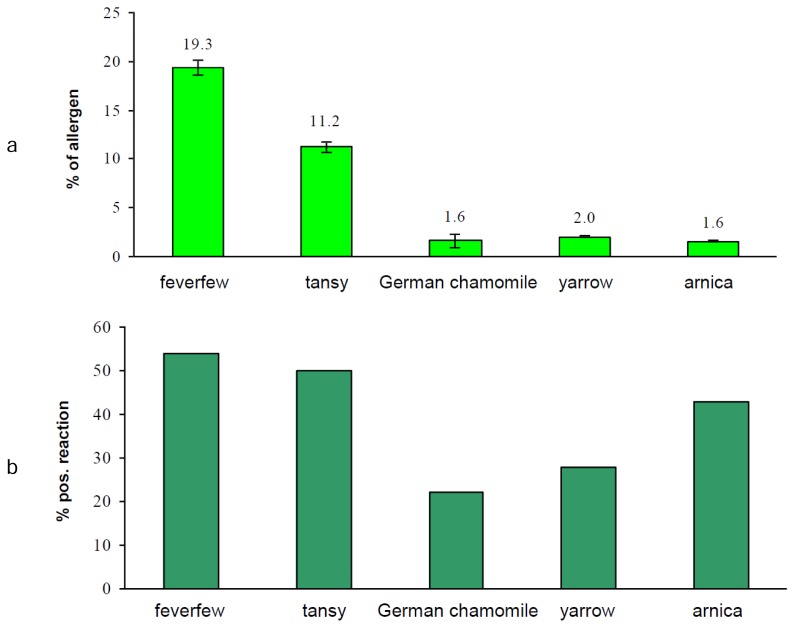
a) % of allergens of individual plant extracts measured by spectrophotometry, calc. as % parthenolide b) % positive reactions among 125 patients with at least one positive reaction to one of the Compositae extracts (see Tab. 2)

For the comparison of the SL contents of the single plant extracts (results of spectrophotometric analyses, Fig. 4) with the results of a clinically conducted patch test with patients prior to the quantification analyses, data from the Information Network of Departments of Dermatology (IVDK) were analyzed (see “Experimental”). All in all, 15,591 patients were patch-tested during the study period. In 1,816 patients, all of the five constituents of the Compositae mix and SSO were patch-tested. Being an emulsifier, SSO may elicit irritant, doubtful, and false-positive patch test reactions. In addition, true allergic reactions to SSO do occur. As SSO was present in all patch test preparations, and this analysis was focused on composite allergies, the data of all the patients with non-negative reactions to SSO (n = 38) were excluded from further analysis. Of the remaining 1,778 patients, 125 reacted positively to at least one the Compositae extracts. Patch test results from these 125 patients are presented in Tab. 2 and Fig. 5b, respectively. The same extracts applied in this study were used for the spectrophotometric analyses presented in this paper.

## Discussion

Using parthenolide (sesquiterpene lactone with an α-methylene-γ-butyrolactone moiety), optimum reaction conditions could be found for the Michael-addition of this compound with sulfhydryl groups. In this way, the reaction of allergenic SLs with proteins eliciting the SL-hypersensitivity could be imitated. L-Cysteine ethyl ester hydrochloride was selected as a source of free sulfhydryl (-SH) groups due to its good solubility in the organic solvents used. SH-groups were present in excess and the amounts of allergenic SLs were easily deduced by calculating the difference of SH-groups before and after addition of the SLs. Parthenolide as a strong allergenic SL [1] was chosen for the method development because of its occurrence in tansy and feverfew extracts and because of its commercial availability. The method was also proven to be well-suited for many other SLs containing an α-methylene-γ-lactone group like alantolactone, isoalantolactone, anthecotulide, and helenaline, occurring in Asteraceae as well [15]. Moreover, costunolide (found in Asteraceae e.g. *Saussurea* and *Artemisia* and Lauraceae e.g. *Laurus*) and dehydrocostuslactone (only present in the family Lauraceae) could be easily quantified using the given spectrophotometric method.

The results of these measurements demonstrate a good recovery and precision of the method within the linear concentration range with no disturbing matrix effects.

Applying this method to plant extracts, it was found that feverfew contains the highest amount of allergenic SLs (19%) compared with the other four plants (see Fig. 4). These results were expected and in accordance with the literature data [1]. On the contrary, even German chamomile, which was expected to exert no allergenic potential, showed a low content of allergens (0.65%). This low content may be due to anthecotulide (previously isolated from *Anthemis cotula*), which is usually not found in *Chamomilla recutita* (German chamomile), but the admixture of *Anthemis cotula* in Chamomile is considered to be responsible for the contamination of the plant material. The percentage of allergenic SLs in tansy, yarrow, and arnica extract was analyzed to be 11%, 2% and 3%, respectively. It is well-known that these plants contain numerous SLs with guaianolide, eudesmanolide, or germacranolide basic structures; the amount of parthenolide in tansy was shown to vary from 0.28–0.51% [1] and 0.06–1.33% [15], respectively.

SSO is a mixture of partial esters of sorbitol [16] used to emulsify extracts for patch testing. The allergenic potential of SSO has long ago been dermatologically confirmed [17]. Surprisingly, SSO reacts with L-Cysteine ethylester hydrochloride to a very small extent under the same conditions, although the α-methylene-γ-lactone partial structure responsible for this mode of reaction is absent.

In order to compare the spectrophotometrically obtained data with the clinical results, the % of allergenic SLs of each extract determined by UV/VIS-spectrophotometry was recalculated to get comparable values as the concentrations used were not the same for each tested extract (see Tab. 2, column “conc.”). Thus, the %-result of arnica was divided by two (equivalent to 0.5%), whereas the percentage of German chamomile was multiplied by 2.5 (equivalent to 2.5%). The results of the other three extracts (feverfew, tansy, and yarrow) were not changed as they were used in a concentration of 1% each for clinical testing.

As illustrated in Fig. 5, the results correlate fairly well with the exception of arnica extract. Those extracts with a higher amount of allergenic SLs determined spectrophotometrically, i.e. feverfew and tansy, showed a higher incidence of allergic patch test reactions, while those with a lower amount of allergenic SLs, i.e. German chamomile and yarrow, elicited significantly fewer allergic patch test reactions (no overlap of the 95%-CIs in Tab. 2). Reactions to arnica may be partly due to other allergens than SLs, e.g. polyacetylenes. This study reports a good coherence between the results obtained by a simple spectrophotometric method and clinical data concerning allergenic SLs in Asteraceae plant extracts for the very first time. It is possible to quantitatively determine allergenic SLs in plant extracts even if they are still unknown. Thus, the introduced method is a valuable tool for evaluating the allergenic potential of Asteraceae extracts and for the simple and efficient quality control of extracts in the Compositae mix.

## Experimental

### Reagents and Solutions

The stock solution of L-Cysteine ethyl ester hydrochloride (CAS 868-59-7; Fluka, Germany) was prepared by dissolving 2.0 mg of the substance in 5.0 ml methanol just before use. The stock solution of parthenolide (CAS 20554-84-1; Sigma, Germany) was prepared by dissolving 3.4 mg parthenolide in 3.0 ml methanol/dichloromethane (1/1, v/v). Stock solutions of santonine (CAS 481-06-1) (3.3 mg/3.0 ml), alantolactone (CAS 546-43-0) (2.7 mg/3.0 ml), anthecotulide (CAS 23971-84-8) (3.4 mg/3.0 ml), costunolide (CAS 553-21-9) (2.3 mg/3.0 ml), dehydrocostuslactone (CAS 477-43-0) (2.2 mg/3.0 ml), helenaline (CAS 6754-13-8) (2.3 mg/3.0 ml), and isoalantolactone (CAS 470-17-7) (2.8 mg/3.0 ml) were prepared in the same way as parthenolide. All SLs were delivered from Hermal (Reinbek, Germany) except santonine (Aldrich, Austria) (chemical structures see Fig. 1). The sodium phosphate dilution buffer (pH = 8.0) was prepared by dissolving 0.074 g NaH_2_PO_4_·H_2_O (CAS 10049-21-5; Merck, Germany) and 1.682 g Na_2_HPO_4_·2H_2_O (CAS 10028-24-7; Merck, Germany) in 100 ml distilled water. The pH values were measured with a pH-meter (ProMega Technik, Austria). Triethylamine (CAS 121-44-8; Sigma, Germany) was used as a pure substance.

The stock solution of Ellman’s reagent (5,5′-Dithiobis[2-nitrobenzoic acid]) (CAS 69-78-3; Sigma, Germany) was prepared by dissolving 16 mg of the reagent in 1 ml sodium phosphate dilution buffer (pH = 8.0).

The individual extracts tested in this work were as follows (plant, part of the plant used for extraction): arnica (*Arnica montana* L.), flowers; German chamomile (*Chamomilla recutita* L. Rauschert), flowers; tansy (*Tanacetum vulgare* L.), herb; yarrow (*Achillea millefolium* L.), herb; feverfew (*Tanacetum parthenium* L.), flowers. All extracts were obtained from Hermal, prepared by short-time carbon dioxide (CO_2_) extraction (approx. 90 sec). The stock solutions of the extracts (2%) were prepared by dissolving 20 mg of each extract in 1 ml methanol/dichloromethane (1/1, v/v).

The stock solution of sorbitan sesquioleate (SSO) (CAS 8007-43-0; Hermal, Reinbek) was prepared by dissolving 20 mg of SSO in 1 ml methanol/dichloromethane (1/1, v/v).

### Solutions for Spectrophotometric Measurements

Test solution 1 was prepared by adding approx. 460 μl of the stock solution of L-Cysteine ethyl ester hydrochloride, approx. 40 μl of triethylamine (molar ratio 1:290), and 10 μl of the stock solution of the extract/pure compound successively in a 10 ml volumetric flask. The solution was gently shaken by hand and left for 30 min. Subsequently 30 μl of the stock solution of Ellman’s reagent was added and the flask was completed to 10 ml with methanol and mixed by hand. After 5 min, the solution was centrifuged with 13,500 rpm for 5 min and the absorption was measured at 412 nm against compensation solution 1 giving absorption A1.

Compensation solution 1 was prepared and measured in the same way as test solution 1, but instead of the stock solution of L-Cysteine ethyl ester hydrochloride, only methanol was added.

Test solution 2 was prepared in the same way as test solution 1, but instead of 10 μl of the stock solution of the extract/pure compound, 10 μl of methanol/dichloromethane (1/1, v/v) were added. Spectrophotometric measurement of this solution at 412 nm against compensation solution 2 gave absorption A2.

Compensation solution 2 was prepared and measured in the same way as test solution 2, but instead of the stock solution of L-Cysteine ethyl ester hydrochloride, only methanol was added.

### Patch Test Material

Five individual plant extracts were applied for the epicutaneous patch test (see Tab. 2). The individually tested extracts had the same percentages as in the Compositae mix, i.e. arnica (0.5%), German chamomile (2.5%), yarrow (1%), tansy (1%), and feverfew (1%), all in petrolatum, each containing 20% SSO as an emulsifier.

### Spectrophotometric Measurement

Absorption was measured with a Beckman DU-640 UV/VIS spectrophotometer with 1 cm cells. The difference of A_2_–A_1_ gave the calculated absorbance A which should be within the linear absorption range (from 0.2 to 1.0), otherwise an appropriate absorption can be achieved by decreasing or increasing the aliquot of the extract solution. The percentage (%) of allergens, expressed as parthenolide (mol weight = 248), can be calculated from the formula given in Fig. 3.

### Patch Testing

The Compositae mix described in the patch test material was part of the baseline of the German Contact Dermatitis Research Group (DKG) series from 2000 to 2006. Its single constituents were part of a DKG plant series. All departments of dermatology forming the Information Network of Departments of Dermatology (IVDK) are DKG members. Patch tests are performed and read according to international and DKG guidelines [12]. In the IVDK headquarters at the University of Göttingen, Germany, patch test data and clinical data of all patients patch-tested in the IVDK are collected and evaluated after strict quality control [13, 14]. Based on the patch test reactions at day 3 (or day 4 in few exceptional cases), we analyzed the patch test results with the single components of the Compositae mix of the period from April 2003 to November 2004. For statistical analysis, SAS 9.1 software (SAS Institute, NC, USA) was used. Differences between disjunct subgroups of data were tested for statistical significance using Fisher’s exact test.

## Figures and Tables

**Tab. 1:** Intra- and interday precision after determination of several parthenolide samples

Concentration of parthenolide (μmol/l)	Intraday precision (% RSD)	Interday precision (% RSD)
11.41	1.30	2.18
22.82	1.99	2.05
68.46	2.85	2.44

**Tab. 2:** Patch test reactions of 125 Compositae-sensitive patients. See text for details.

Extract	conc.	neg	?	+	++	+++	ir	% pos (95%-CI)
Arnica flowers	0.5%	68	3	30	16	8	0	43% (34–52%)
German chamomile flowers	2.5%	96	2	18	6	3	0	22% (15–30%)
Tansy herb	1.0%	61	2	35	17	10	0	50% (41–59%)
Yarrow herb	1.0%	84	6	26	6	3	0	28% (20–37%)
Feverfew flowers	1.0%	54	4	34	22	11	0	54% (44–63%)

All test preparations were applied in petrolatum.

conc. = concentration; neg. = negative; ? = questionable; severity of allergic reaction increases with increasing number of “+”; ir = irritant; % pos. = percentage positive; 95%-CI = 95%-confidence-interval.
